# Brain Activity during Different Throwing Games: EEG Exploratory Study

**DOI:** 10.3390/ijerph17186796

**Published:** 2020-09-17

**Authors:** Alfonso García-Monge, Henar Rodríguez-Navarro, Gustavo González-Calvo, Daniel Bores-García

**Affiliations:** 1 Department of Didactics of Musical, Artistic and Body Expression, Faculty of Education of Valladolid, University of Valladolid, 47011 Valladolid, Spain; alfonso.garcia.monge@uva.es; 2 Department of Pedagogy, Faculty of Education of Valladolid, University of Valladolid, 47011 Valladolid, Spain; henarrod@pdg.uva.es; 3 Department of Didactics of Musical, Artistic and Body Expression, Faculty of Education of Palencia, University of Valladolid, 34004 Palencia, Spain; gustavo.gonzalez@uva.es; 4 Department of Physical Therapy, Occupational Therapy, Rehabilitation and Physical Medicine, Faculty of Health Sciences, Rey Juan Carlos University, Alcorcón, 28922 Madrid, Spain

**Keywords:** EEG, play, physical games, throwing games, beta oscillations

## Abstract

The purpose of this study is to explore the differences in brain activity in various types of throwing games by making encephalographic records. Three conditions of throwing games were compared looking for significant differences (simple throwing, throwing to a goal, and simultaneous throwing with another player). After signal processing, power spectral densities were compared through variance analysis (*p* ≤ 0.001). Significant differences were found especially in high-beta oscillations (22–30 Hz). “Goal” and “Simultaneous” throwing conditions show significantly higher values than those shown for throws without opponent. This can be explained by the higher demand for motor control and the higher arousal in competition situations. On the other hand, the high-beta records of the “Goal” condition are significantly higher than those of the “Simultaneous” throwing, which could be understood from the association of the beta waves with decision-making processes. These results support the difference in brain activity during similar games. This has several implications: opening up a path to study the effects of each specific game on brain activity and calling into question the transfer of research findings on animal play to all types of human play.

## 1. Introduction

The words “play” or “game” capture a wide range of meanings, activities, and behaviors, and their definition remains a controversial issue [1]. Several studies, mainly conducted with animals [2], have delved into the neurophysiological basis of play. Although neurophysiological studies abound in sports and e-gaming, there is still little research on children’s physical games. This study is intended as an initial attempt to investigate the brain processes that occur in different types of games. This work expands our line of research on children’s play [3,4] by analyzing electroencephalographic activity during play episodes. The objective of this research is to know the differences in brain activity during different types of throwing games by taking encephalographic (EEG) records.

### 1.1. Brain Wave Indicators

Neural activity causes signals of different frequencies. By measuring electrical activity of neuronal assemblies with millisecond temporal resolution, EEG offer the possibility of studying brain function in real time. Unfortunately, the spatial resolution afforded by EEG is constrained by several factors [5].

Studies through electroencephalography (EEG) have associated different frequencies with types of brain activity. Existing research is extensive, and, in this section, we will focus on those contributions close to our subject of study. We will first summarize some characteristics associated with each type of frequency and then we will focus on studies on brain activity related to emotions and motor skills.

In the following section, a brief review of various EEG bands and their functional roles will be presented. EEG oscillations are traditionally subdivided into five frequency bands: delta (0.5–4 Hz), theta (4–7 Hz), alpha (7–13 Hz), beta (13–30 Hz), and gamma (30–50 Hz). The functional role of these frequencies is still debated, given the contextual variations in which EEG is gathered [6].

#### 1.1.1. Delta Band (0.5–4 Hz)

Delta rhythms reflect low-frequency activity (1–4 Hz) and are associated with stages of deep sleep [7]. The role of the oscillation delta between frontal and parietal zones has been observed in decision-making processes [8], auditory attention, and memory updating [9]. The coupling between the beta band and the delta band has been related to the temporal prediction of events and the accuracy of the elaboration of a response [10]. *Delta is also the predominant activity in infants during the first two years of life and slow delta and theta activity diminish with increasing age, whereas the faster alpha and beta bands increase almost linearly across the life span* [5] (59).

#### 1.1.2. Theta Band (4–7 Hz)

Theta activity refers to EEG activity within the 4–7 Hz range, prominently seen during sleep. During wakefulness, two different types of theta activity have been described in adults. The first shows a widespread scalp distribution and has been linked to decreased alertness (drowsiness) and impaired information processing [11]. The second, the so-called frontal midline theta activity, is characterized by a frontal midline distribution and has been associated with focused attention, mental effort, and effective stimulus processing [5]. In children it is common to find a greater presence of this band, which decreases over time until they reach adolescence [12].

Theta is believed to enable the coding and decoding of hippocampal learning in the neocortex, especially the frontal lobes [13]. They would have an important role in cognitive processing, memory performance, and learning mechanisms. The theta front midline has been associated with good cognitive control during planning [14,15], and as an indicator of optimal attentional engagement during skilled putting performance [16].

#### 1.1.3. Alpha Band (7–13 Hz)

The alpha rhythm refers to EEG activity within the 7–13 Hz range and can be easily recorded during states of relaxed wakefulness. During normal development, an alpha frequency of 8 Hz appears at the age of three and remains stable for the rest of life [5].

The alpha rhythm has been considered as a means of communication between the thalamus and the cerebral cortex [17]. This oscillation between both structures appears with closed eyes, especially in occipital areas of the cortex. When the eyes are opened, the activity alpha disappears and is replaced by a much more unsynchronized activity within the bandwidth of beta, which is related to sensory or motor processing. Attentional processing or cognitive tasks attenuate the alpha waves [18]. *In cognitive tasks, lower alpha (e.g., 8–10 Hz) desynchronization (suppression) has been associated with stimulus-unspecific and task-unspecific increases in attentional demands* [5] (60). Klimesch [19] suggested that only slower alpha frequencies reflect attention characteristics such as alertness and expectancy.

Another oscillatory activity within the frequency band alpha is the mu (8-13 Hz) or sensorimotor rhythm (SMR), its waves are sharper and less sinusoidal, it is also known as precentral or rotary activity alpha.

#### 1.1.4. Beta Band (13–30 Hz)

Traditionally, lower-voltage oscillations within the 13–30 Hz frequency range have been referred to as beta. In adults, beta activity has amplitudes between 10 and 20 µV (microvolts), presents mainly a symmetrical front-central distribution and typically replaces alpha rhythm during cognitive activity [5]. Beta activity normally increases during drowsiness, light sleep, and with mental activation [7]. The frontal and central regions of the brain are locations where enhanced beta waves can be observed during activeness, anxious thinking, problem solving, and deep concentration [18]. Evoked oscillatory beta activity was associated with projections from sensory-specific cortex and higher beta responses were reported during multisensory stimuli in comparison with unisensory stimuli [20].

#### 1.1.5. Gamma Band (30–50 Hz)

Gamma Waves (above 25 Hz) are fast oscillations and are usually found during conscious perception. Gamma oscillations have been associated with attention, arousal, object recognition, learning, preparing for a move, top-down modulation of sensory processes, and, in some cases, perceptual binding [21,22]. The work of Martini et al. [23] shows that in the face of unpleasant stimuli, gamma waves appear.

It has been proposed that the low frequency bands (e.g., delta and theta) may reflect the activity of motivational and emotional systems, while the higher frequency bands (e.g., alpha and beta) have been more involved in inhibitory processes [24,25].

In any case, there are many contextual and personal variables. For example, Tran, Craig, and McIsaac [26] showed how alpha values were higher in extroverted people than in introverted ones, and [27] point out that agreeableness is associated with theta activity in frontal and occipital lobes; neuroticism is detected with theta activity in parieto-temporal lobes; and extroversion is associated with alpha and theta frequency bands in frontal and temporal lobes.

### 1.2. Motricity and Cortical Records

Park, Fairweather, and Donalson [28] pointed out that EEG research within the sporting context has largely focused on alpha rhythms involved in the inhibition of unnecessary or conflicting processing in the cortex, global arousal, and attentional processes. Babiloni et al. [29] showed that in the eyes-closed resting state the alpha waves in the parietal and occipital areas were higher in expertise athletes. Del Percio et al. [30] registered a reduced alpha desynchronization over the motor cortex during preparation of movements in expertise athletes. High alpha activity also in the left temporal lobe is related to better performance, but the poorest shots during archery performance were associated with the highest levels of both temporal lobes’ EEG alpha power [31]. A better performance was associated with an increase in upper alpha power at parietal electrodes, along with an increase in theta power at frontal electrodes [32]. Deeny et al. [33] showed that expert marksmen exhibited lower coherence between left temporal (T3) and mid-line frontal (Fz) regions for low-alpha and low-beta frequencies, lower coherence for high-alpha between all left hemisphere sites and (Fz), and lower coherence between T3 and all midline sites for the low-beta band (experts engage in less cortico-cortical communication, which implies decreased involvement of cognition with motor processes).

In general, higher levels of intelligence and superior performance have been associated with reduced cortical activation [34,35].

A recent review [36] concluded that among the electroencephalographic components examined, only sensorimotor rhythm (8–13 Hz oscillation in the sensorimotor cortex, related to the regulation of cognitive-motor information processing in motor performance) demonstrated a consistent and causal relationship with superior precision motor performance.

On the other hand, focusing on child populations, the relationship between brain development and the acquisition of motor skills is a developing field [37]. The structure of the brain changes rapidly throughout childhood, and it is a challenge to separate the contributions of specific neural changes from development and learning [38]. Electroencephalographic measurements of movement detected that up to the age of 8–9 years, there is no slow negative change prior (“readiness potential”) to movement [37].

### 1.3. Emotion and Cortical Records

Theta assigns play an important role in human emotional processing and in general excitation processes [39]. Theta has also been explicitly involved in emotional brain networks [40].

In the review by Shu et al. [41] they point out that alpha values are high in the face of emotions of anger, anxiety, amusement, happiness, and joy, and low in cases of fear. Beta values are high in situations of amusement. Dzedzickis, Kaklauskas, and Bucinskas [42], in their review on emotion recognition through EEG, show that beta waves appear associated to an alert or anxious state. Also, negative emotions are related to increased beta responses in humans [43].

Some studies also have reported connections between high-frequency-band (>30 Hz) activities and emotions [44,45]. Matsumoto et al. [46] suggested that these high-frequency-band activities reflect emotional processing, playing an important role in the cognitive control of emotions [47]. Abhang et al. [48] point out that high beta waves (18–40 Hz) are associated with significant stress, anxiety, paranoia, high energy, and high arousal.

In any case, studies such as those by Wei et al. [49] warn of personal differences given the neural circuit underlying emotional process influencing on personality.

### 1.4. Children’s Play

As Koeners and Francis propose [1], defining play is an open question and lively debate, but many authors, as Stevens [50] summarized, propose that play is an “altered state” related to fun and the state of flow proposed by Csikszentmihalyi, recognizing that this is the essence of the play experience; Huizinga’s “intense and utter” absorption. Authors such as Moyá [51] associate that “altered state” with waves between 10 and 12 Hz (alpha oscillations).

Probably, the core neural circuitry that motivates an animal to engage in playful social interactions is shared among mammalian species that engage in play [52]. Although the game circuits reside predominantly in subcortical structures such as the hypothalamus, the striatum, or the amygdala [52], different empathic or tactical aspects are associated with different areas of the cortex, especially prefrontal areas [52]. Play is usually associated with emotions of joy, regulated by subcortical limbic networks, and associated with increases in dopamine levels [53] linked to mental shifting, creativity, and motivation, but may also produce stress, frustration, and addiction [23]. As Koeners and Francis propose [1], this ambivalence is consistent with Sutton Smith’s approach that the exciting and rewarding aspects of the game are often found in the ambiguity between creation and destruction [54]. These emotional aspects would be reflected in an increase in theta waves during play [55,56], although with great personal differences possibly due to each player’s different approach to play [3] or the play type [4].

## 2. Materials and Methods

### 2.1. Participants

To calculate the sample size, the biomath application (http://biomath.info) was used by performing a t-test with significance level: alpha = 0.05 and power of sample size = 0.90 was calculated from the mean and standard deviation of the study data. The following formula [57] was applied to confirm the result of *t*-test:

(1) $$sample\;size=\frac{Z^{2}*SD^{2}}{d}$$

where “Z” is standard normal variate (at 5% type 1 error (*p* < 0.05) it is 1.96); “SD” is the standard deviation of variable; and “d” the absolute error or precision (0.05 in this case). The result (<8) is common in preliminary EEG studies [58,59,60,61,62]. A total of eight children volunteers (four males and four females, mean age 7.20 years ± 0.19) participated in the experiment. All the participants were right-handed and healthy. All participants and their families gave written informed consent. The study was performed in accordance with the Declaration of Helsinki. The experiment was accompanied by an educational activity for participants on the functioning of the brain and the recording of brain signals. The participants and their families have been receiving reports on the results obtained from the different analyses of the data.

### 2.2. Procedure

The room that was set up for the sessions was isolated in order to avoid any kind of distraction or noise. Participants sat in a comfortable chair with their arms resting on the launch table.

**Figure 1:**
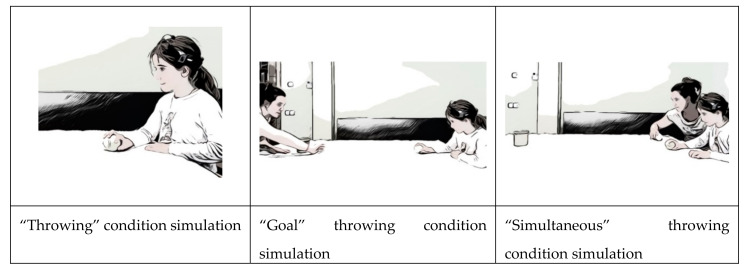
Simulation of three experimental conditions (“Throwing,” “Goal,” and “Simultaneous”).

Three throwing games were proposed to the participants (Figure 1):

- First condition: “Throwing.” Participant had to throw tennis balls at 10 wooden pieces from 2.5m. In preliminary tests we had seen that it was an easy challenge for children of this age.
- Second condition: “Goal.” Participant had to throw, from a distance of 2.5m, tennis balls to a goal (of 80cm) defended by a dummy handled by a friend of the participant. This challenge increased the complexity of the throw as the target became changeable and a relational variable was introduced into the game.
- Third condition: “Simultaneous.” This consisted of a throw to 10 wooden blocks located 2.5 m away, simultaneously to another opponent who threw to the same targets. This challenge introduces a time factor (knocking down the blocks before the opponent) and therefore could increase the arousal.

“Throwing” was proposed as the first activity to serve as a throwing test. The “simultaneous” throwing challenge was left for the end since it was assumed that it would generate the highest excitement and it was intended that this possible state would not influence a later challenge. The experiments were carried out between 5 and 6 pm. In each game they were able to perform 15 throws. We did not leave more attempts to avoid disinterest in the task and because in experiments with children it is recommended to use an electrode application time under 30 [63].

After a brief explanation of the procedure and instructions to minimize movement and speech during the recording, the EEG recording system was put in place. An Emotiv EPOC^®®®®^ headset with 16 electrodes, 14 EEG recording channels (AF3, AF4, F3, F4, F7, F8, FC5, FC6, P7, P8, T7, T8, O1, and O2) and 2 reference electrodes (P3 and P4), positioned according to the International System 10–20, was used. The electrodes of this system are contact and saline type. The Emotiv Control Panel software provides visual monitoring of the electrode impedance lower than 5 kΩ (kilo-ohmios) in order to obtain a good quality signal. The recorded EEG signal, with a sampling frequency of 128 Hz, is sent wirelessly to a Bluetooth receiver placed on the computer. The Emotiv EPOC^®®®®^ has an artifact cancelation system on its reference electrodes and a filter for the frequencies 50 (notch filter) and 60 Hz. Emotiv-epoc has been widely used in studies on emotion detection [64,65,66] or in movement situations [67,68].

### 2.3. Signal Pre-processing

**Figure 2:**
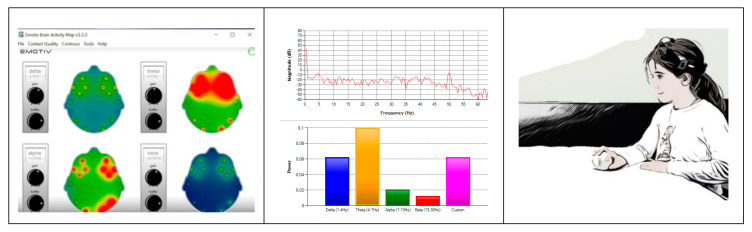
Examples of Emotiv Brain Activity Map and TestBench applications interfaces in conjunction with a video image simulation.

For a first inspection of the data the Emotiv Brain Activity Map (v3.3.3) and Emotiv TestBench (v1.5.0.3) (Emotiv, San Francisco, CA, USA) applications were used. The Emotiv Brain Activity Map shows brain power activity maps at different frequencies obtained through a spectral analysis (Fast Fourier Transform—FFT) of each channel signal. The Emotiv TestBench displays the spectrum of the signals through a FFT (in decibels –dB-). In this first inspection, brain maps were compared with the spectrum and video images of each participant’s actions in order to identify events (Figure 2).

**Figure 3:**
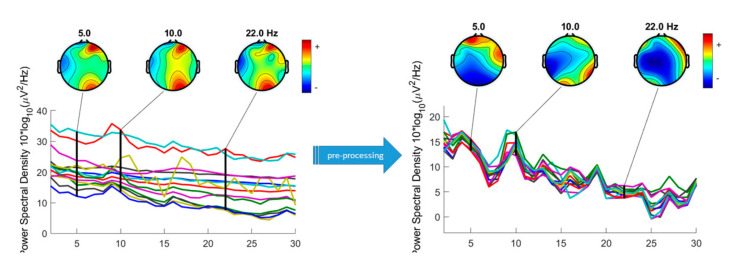
Pre- and Post-processed channels spectra.

Data pre-processing and analyses were carried out using EEGLAB toolbox (v.2019.1) (Swartz Center for Computational Neuroscience, La Jolla, CA, USA) for Matlab (MathWorks, Natick, MA, USA). Baseline of the EEG signal for each channel was removed. A spatial filtering of Common Average Reference (CAR) was applied. For frequential filtering, data were high-pass filtered at 1Hz to remove slow drifts. Artefacts were visually identified and rejected from the channels data (Figure 3).

**Figure 4:**
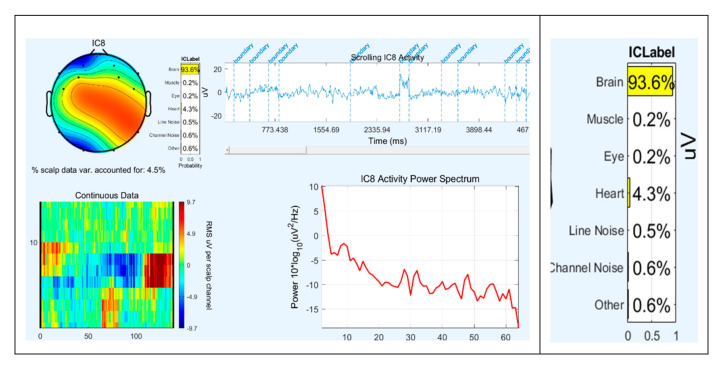
Example of the EEGLab ICALabel interface showing the properties of each component.

Data were decomposed by Independent Component Analysis (ICA). Components that did not account for brain were visually identified and removed. For this purpose, ICALabel tool (an electroencephalographic independent component classifier) was used (Figure 4). This is a plugin that, among other things, shows us the probability that the component picks up brain activity or other artefacts (muscles, blinking, heart, etc.).

### 2.4. Statistical Analysis

The frequency domain analysis was performed using the Fast Fourier Transform (FFT) algorithm (with the resolution of 0.125 Hz) to calculate absolute (µV^2^/Hz) power spectral density within theta (4–7 Hz), alpha (7–13 Hz), and beta (13–30 Hz) bands (this is a power-based logarithmic transform based on the microvolt (µV) measurement and the time, calculated for each frequency band). Channels and component measurements were pre-compute. Power spectral density metrics for each condition were calculated.

EEGLAB allows users to use either parametric or non-parametric statistics to compute and estimate the reliability of these differences across conditions (“throwing,” “goal,” and “simultaneous”). The toolbox also allows the obtaining of different spectrum parameters such as the maximum and minimum, mean, medium, mode, standard deviation, and range.

EEGLAB allows performing analysis of variance on power spectra. For mean power spectra, the p-values are computed at every frequency. In this case an analysis of variance (ANOVA) test was developed in order to detect statistical differences between the three conditions. The specific time-frequency point was considered significant at *p* ≤ 0.001. EEGLab designers recommend that while parametric statistics might be adequate for exploring data, it is better to use permutation-based statistics to plot final results.

## 3. Results

### 3.1. Preliminary Data Inspection

For a first inspection of the data the Emotiv Brain Activity Map application (v3.3.3) and the Emotiv TestBench (v1.5.0.3) applications were used. The maps are generated by performing a spectral analysis for each of the 14 sensors, then dividing the sensor readings into delta, theta, alpha, and beta oscillations. The Emotiv TestBench show the spectrum averaged topographies (based on FFT) of the signals. The brain maps and the FFT were viewed simultaneously with the video recordings of the participants’ actions in order to identify events.

**Figure 5:**
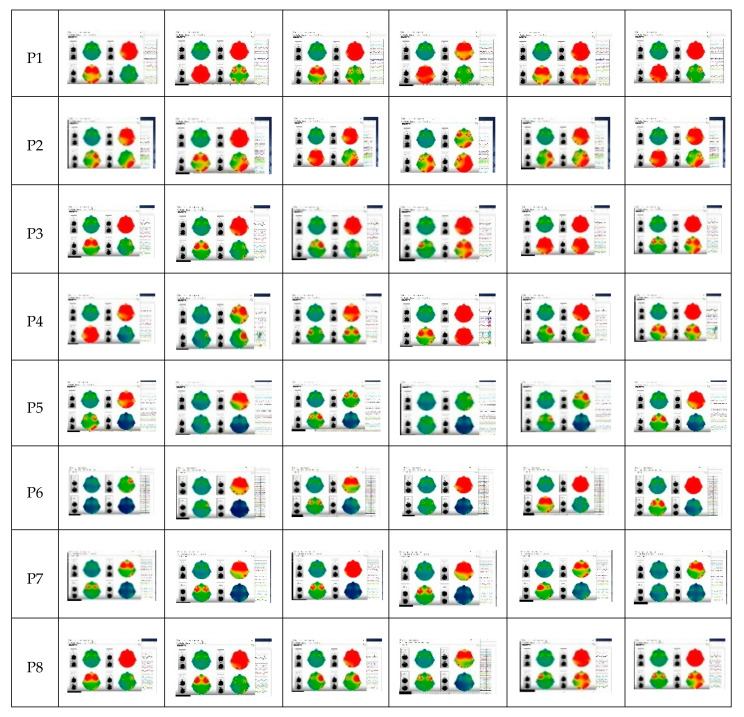
Brain activity sequences of eight participants (one line for each participant) in the “throwing” condition. Each frame shows the delta (upper-left), theta (upper-right), alpha (bottom-left), and beta (bottom-right) oscillations of each moment. These frames, taken every two seconds, correspond to the central 12” of the task.

In the condition of throwing tennis balls at 10 wooden pieces from 2.5 m (“throwing”), an increase in theta and beta waves was seen in six of the participants after knocking down some pieces (especially after a successful shot after several failed attempts). Also increases in theta (75 dB) and mid-beta (20 dB) waves when throwing to the last piece of wood were noticed. In general, high fluctuating theta values (41 to 78 dB), moderate alpha values (especially associated with frontal and right lobe areas), and contained beta wave values (-30 to 15 dB) were observed (Figure 5).

**Figure 6:**
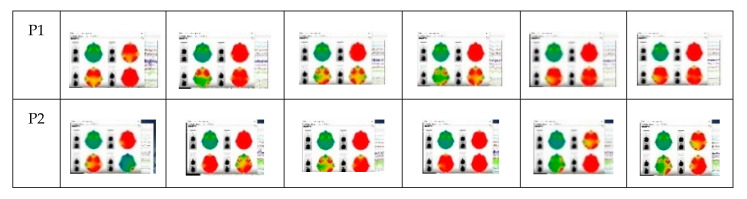

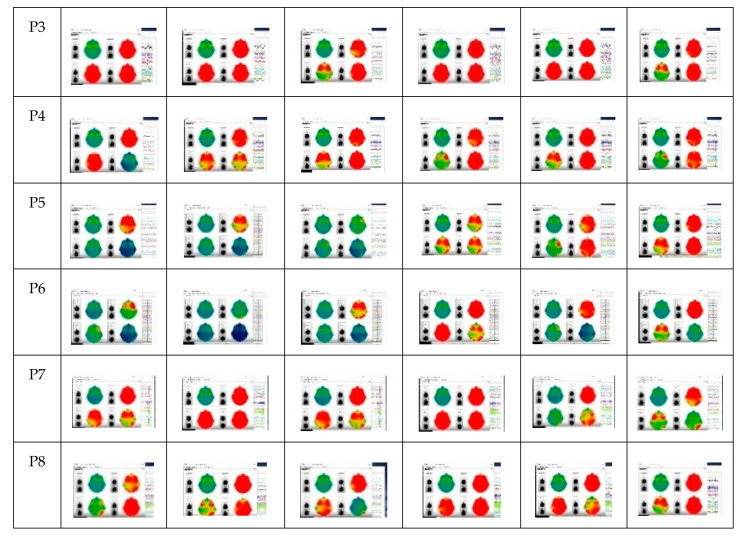
Brain activity sequences of eight participants (one line for each participant) in the “goal” throwing condition. Each frame shows the delta (upper-left), theta (upper-right), alpha (bottom-left), and beta (bottom-right) oscillations of each moment. These frames, taken every two seconds, correspond to the central 12” of the task.

In the throwing condition (from a distance of 2.5 m) of tennis balls to a goal (80 cm) defended by a dummy handled by a friend of the participant (“goal”), a progressive increase of theta values was observed throughout the experiment, as well as a constant fluctuation of the alpha oscillations in the frontal areas (-9 to 11 dB), and a high value of the beta waves (up to 15 dB). In three cases, an increase in theta was observed after the failures. In four cases after scoring goals, increases in theta and beta were observed. Five children paused their play and attempted throwing feints. In those cases, decreases in theta activity and records of beta oscillations in occipital areas were observed (Figure 6).

**Figure 7:**
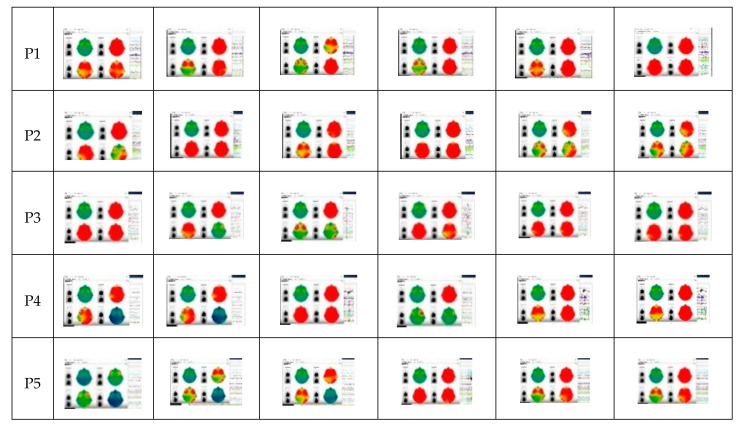

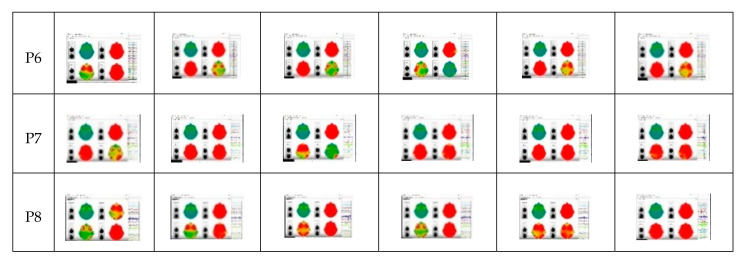
Brain activity sequences of eight participants in the “simultaneous” throwing condition. Each frame shows the delta (upper-left), theta (upper-right), alpha (bottom-left), and beta (bottom-right), oscillations of each moment. These frames, taken every two seconds, correspond to the central 12” of the task.

The third condition (“simultaneous”) consisted of a throw at 10 wooden blocks located 2.5 m away, while another opponent threw a ball at the same targets simultaneously. High theta (73–81 dB) values were seen in all participants throughout the game (Figure 7). Alfa showed constant synchronizations and desynchronizations and beta exhibited medium (9 to 14 dB) and high values (up to 15 dB). In three cases a decrease in theta and beta activation was observed in the last throws.

### 3.2. Comparison of the Three Conditions

**Figure 8:**
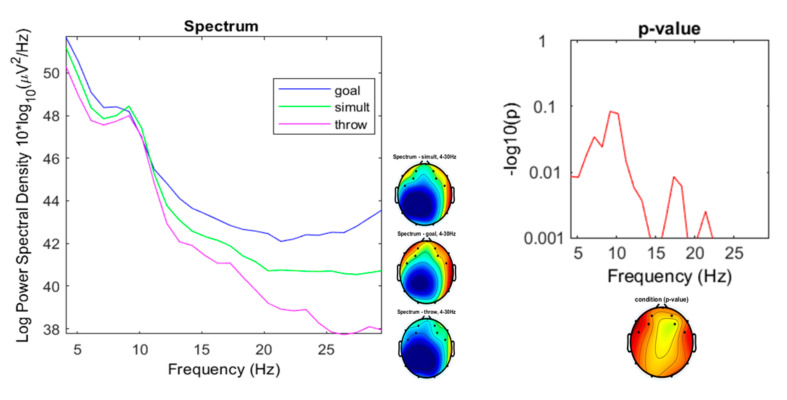
Spectrum plotting of the three conditions components (“throwing,” “goal,” “simultaneous”), with their plot averaged topography over frequency range, and the *p*-value plot for frequencies from 4 to 30 Hz.

The results after the analysis of variance show significant differences (*p* < 0.001) between the power spectral densities (PSD) in the beta oscillations, especially in the high beta as summarized in Figure 8.

**Figure 9:**
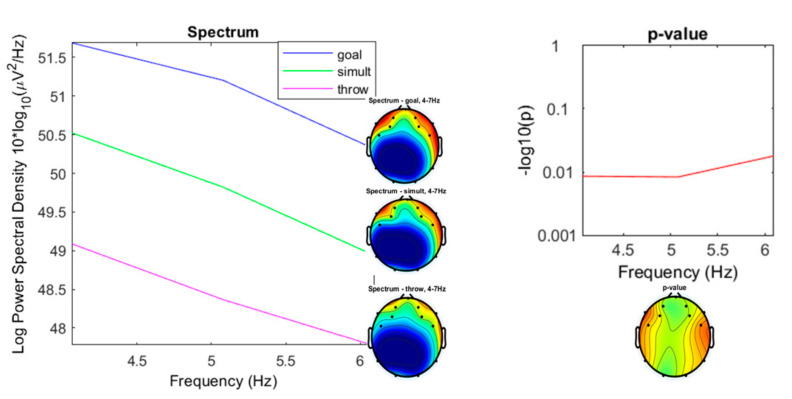
Spectrum plotting of the three conditions components (“throwing,” “goal,” “simultaneous”), with their plot averaged topography over frequency range, and the *p*-value plot for frequencies from 4 to 7 Hz (Theta).

Theta frequencies did not differ significantly (Figure 9). The range of densities for the “throwing” condition was from 47.77 to 49.09 µV^2^/Hz (*M* = 48.41; sd = 0.6603); from 50.31 to 51.69 µV^2^/Hz for the “goal” condition (*M* = 51.07; sd = 0.6967); and 48.94 to 50.52 µV^2^/Hz (*M* = 49.76; sd = 0.7956) for the “simultaneous” condition.

**Figure 10:**
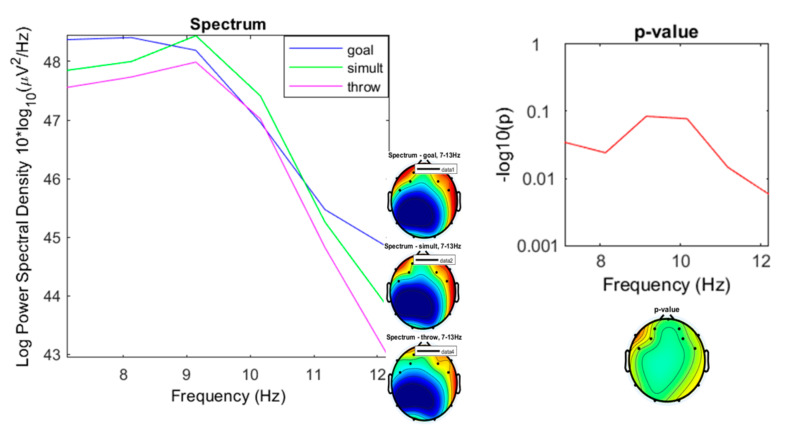
Spectrum plotting of the three conditions components (“throwing,” “goal,” “simultaneous”), with their plot averaged topography over frequency range, and the *p*-value plot for frequencies from 7 to 13 Hz (alpha).

In the alpha band the spectral densities were more similar (Figure 10), with ranges of 42.93 to 47.99 µV^2^/Hz (*M* = 46.34; sd = 2.026) for the “throwing” condition, from 44.81 to 48.41 µV^2^/Hz (*M* = 47.04; sd = 1.574) for the “goal” condition, and from 43.77 to 48.44 µV^2^/Hz (*M* = 46.79; sd = 1.853) for the “simultaneous” condition.

**Figure 11:**
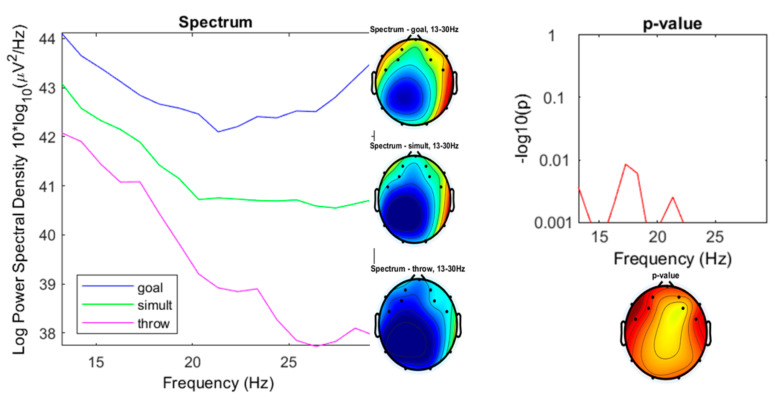
Spectrum plotting of the three conditions components (“throwing,” “goal,” “simultaneous”), with their plot averaged topography over frequency range, and the *p*-value plot for frequencies from 7 to 13 Hz (beta).

Beta oscillations represented the most significant differences (Figure 11), especially in the high-beta band (22–30 Hz).

**Figure 12:**
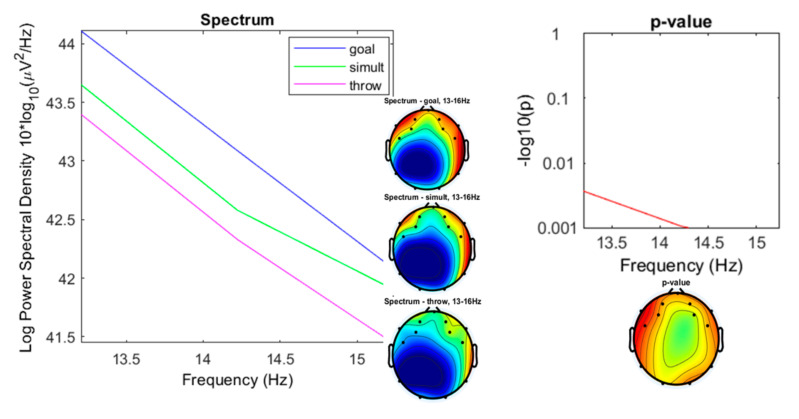
Spectrum plotting of the three conditions components (“throwing,” “goal,” “simultaneous”), with their plot averaged topography over frequency range, and the *p*-value plot for frequencies from13 to 16 Hz (low-beta).

In the spectral densities for low beta (13–16 Hz) the “goal” and “simultaneous” conditions showed their most significant differences (*p* < 0.001) with respect to the “throwing” condition between 14.38 and 16 Hz (Figure 12).

**Figure 13:**
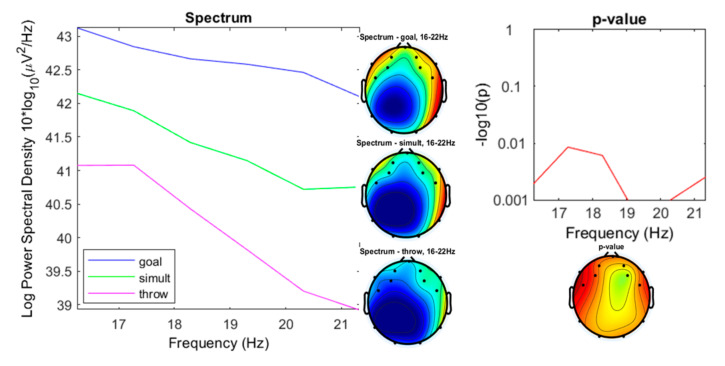
Spectrum plotting of the three conditions components (“throwing,” “goal,” “simultaneous”), with their plot averaged topography over frequency range, and the *p*-value plot for frequencies from16 to 22 Hz (mid-beta).

The mid-range beta (16–22 Hz) spectral densities expressed their most significant differences between 19 and 20.2 Hz (see detail in Figure 13), with ranges for the “throwing” condition between 38.92 to 41.08 µV^2^/Hz (*M* = 40.13; sd = 0.9265), for the “goal” condition from 42.1 to 43.13 µV^2^/Hz (*M* = 42.63; sd = 0.3481), and from 40.72 to 42.15 µV^2^/Hz for the “simultaneous” condition (*M* = 41.35; sd = 0.588).

**Figure 14:**
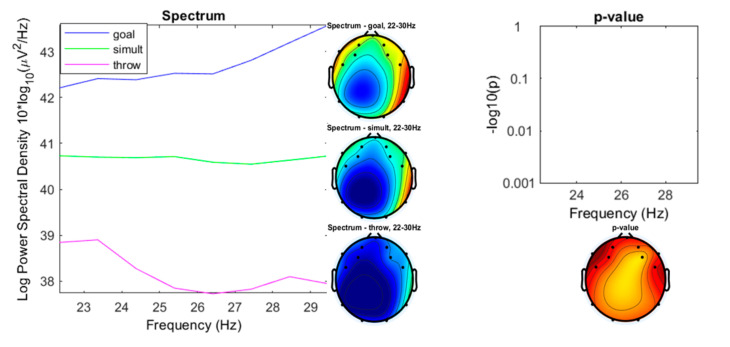
Spectrum plotting of the three conditions components (“throwing,” “goal,” “simultaneous”), with their plot averaged topography over frequency range, and the *p*-value plot for frequencies from 22 to 30 Hz (high-beta).

As mentioned above, the most significant differences between the three experimental conditions were shown in the spectrum range of the high-beta oscillations between 22 and 30 Hz (Figure 14), with ranges for the “throwing” condition from 37.73 to 38.9 µV^2^/Hz (*M* = 38.18; sd = 0.4583), for the “goal” condition from 42.21 to 43.57 µV^2^/Hz (*M* = 42.7; sd = 0.4631), and from 40.55 to 40.73 µV^2^/Hz for the “simultaneous” condition (*M* = 40.67; sd = 0.06847).

## 4. Discussion

EEG signals have been widely used for studying different cognitive functions. In this study we explore the differences in brain activity in three types of throwing games (simple throwing, throwing at a goal, and simultaneous throwing with another player) by taking encephalographic records. The differences, especially in high-beta power, were supported by results. Furthermore, the “throwing” condition presented low values in the beta spectrum compared to the conditions of “goal” and “simultaneous” throwing.

Since the “throwing” condition presented an uncomplicated task for the participants and the association of theta waves with states of emotional excitement [39], a significant difference was expected in this frequency band between the “throwing” condition and those of “goal” and “simultaneous” (in principle, with greater emotional demand given the added uncertainty of throws and interaction with an opponent). The preliminary data inspection of the spectrum averaged topographies showed some difference, but the subsequent statistical analysis confirmed that this difference was not significant. Perhaps this can be due to the short duration of each game (less than 5 min) as indicated by studies based on video games [69].

In the alpha spectrum, between 9 and 11 Hz, there is an increase in the values in the “simultaneous” condition compared to the other two conditions that could be explained by a higher happiness and joy following the analysis of Shu et al. [41], although the difference is not significant. On the other hand, according to Reuderink et al. [70] a significant increase in alpha power range, associated with increasing emotional arousal, could be expected, but is not reflected in this study (there is a non-significant difference in favor of the “simultaneous” condition in the range of mid-alpha, 9–10.5 Hz).

The results show a difference (not significant) in the low-alpha range (7–8 Hz) that may be associated with the higher socio-cognitive processing demanded in the “goal” condition [71]. A larger sample would be needed to confirm this trend.

Beta oscillations are related to demands on the motor and somatosensory cortex [72], as well as to top-down control [73]. Since the conditions of “goal” throwing (in which the target is changing) and “simultaneous” throwing (in which there is a rush to throw) are more exigent, the difference in the beta spectrum versus the “throw” condition could be explained in this sense.

On the other hand, Abhang et al. [74] point out that high beta waves are associated with significant stress, anxiety, high energy, and high arousal, and Dzedzickis, Kaklauskas, and Bucinskas [42] associated them to an alert or anxious state. According to these studies, it is plausible to find less arousal in the less demanding perceptual and relational “throwing” condition.

This record of “controlled stress” in play situations would be in line with studies on the neurophysiology of play such as those by Wang and Aamodt [74]. According to these authors, play activates the brain’s reward circuitry but not negative stress responses, which can facilitate attention and action.

The high values of high-beta in the “goal” condition versus the “simultaneous” throwing condition could be understood from the association of the beta waves with decision-making processes [75,76]. Spitzer and Haegens [77] report that lateralized beta activity during decision-making tasks reflect a dynamic process of accumulatively updating a motor plan. In addition, it appears that beta oscillations are also involved in the timing [10]. Compared to the “goal” condition, the “simultaneous” throwing condition, in theory, involves less decision making (the challenge does not involve the evaluation of the opponent’s reaction) and less timing (the “goal” throwing challenge involves an adjustment to the goalkeeper’s movements).

Versus video games studies that indicate low-beta frequency as the most informative band for discriminating among gaming conditions [78], in this study on physical games, it is the high-beta frequency that shows the differences between variants of the throwing games.

Beta oscillations are also associated with unexpected positive rewards [79], but in this study it is not clear if this indicator can play a role in the results of the different conditions.

As many studies in the field of motor praxeology point out [80], each game introduces players to a particular logic and each modification in the game structure results in different experiences. Although these results need to be confirmed with larger samples and other types of games, they reveal important implications within the field of physical education and, in general, in the study of human play. In the field of physical education, a line of research is opening up that could identify the brain demands of the different tasks proposed. As for the field of human play, it is questionable to transfer the conclusions of studies on animal play to human play (given that these studies are frequently used to make this type of extrapolation [53]) and it raises the question of whether speaking about “play” in general can be meaningful.

This opens up many questions for future research. Further studies would be needed to determine the role of different explanations given to high beta (motor demand of the task, arousal, making decision or reward).

If each game causes different states, can we talk about “play” in general? To get closer to the answer to this question, we can compare brain activity in different situations (relaxation, reading, arithmetic calculation) with records of different play activities such as those proposed in this work.

In addition to the fact that each type of game causes different experiences, are the differences in brain states between players within the same game significant? To solve this problem, we should increase the sample size and try to find different clusters.

## 5. Conclusions

The results show the differences in brain activity between games belonging to the same family (throwing games) but with different characteristics. The main differences between the three compared game situations are seen in the high-beta frequency (20–30 Hz). “Goal” and “simultaneous” throwing conditions show significantly higher values than those shown for throws without opponent. This can be explained by the higher demand for motor control and the higher arousal in competition situations. On the other hand, the high-beta records of the “goal” condition are significantly higher than those of the “simultaneous” throwing, which could be understood from the association of the beta waves with decision-making processes. There are also differences (although not significant) in the theta and alpha waves that would need future studies with larger samples to be confirmed.
